# Exceptional Response in BRAF p.V600E-Mutant Enteric-Type Adenocarcinoma of the Lung With Cutaneous Spread: A Case Report

**DOI:** 10.1016/j.jtocrr.2023.100597

**Published:** 2023-11-02

**Authors:** Marco Sposito, Ilaria Mariangela Scaglione, Serena Eccher, Luca Pasqualin, Alice Avancini, Chiara Colato, Paolo Rosina, Michele Simbolo, Anna Caliò, Aldo Scarpa, Michele Milella, Sara Pilotto, Lorenzo Belluomini

**Affiliations:** a Section of Innovation Biomedicine - Oncology Area, Department of Engineering for Innovation Medicine (DIMI), University of Verona and University and Hospital Trust (AOUI) of Verona, Verona, Italy; b Department of Neurosciences, Biomedicine and Movement Sciences, University of Verona, Verona, Italy; c Section of Pathology, Department of Diagnostic and Public Health, University of Verona, Verona, Italy; d Section of Dermatology and Venereology, Department of Medicine, University of Verona, Verona, Italy

**Keywords:** Non–small cell lung cancer, BRAF p.V600E, Cutaneous metastases, Enteric-type lung adenocarcinoma, Dabrafenib plus trametinib, Case report

## Abstract

**Background:**

Enteric-type adenocarcinoma of the lung (lung-ETAC) is a rare form of lung cancer with histologic similarities to colorectal cancer, with aggressive behavior and unfavorable prognosis.

**Case Presentation:**

An 81-year-old man presented with discolored skin lesions on the chest and abdomen. After comprehensive evaluation, including skin biopsy and molecular profiling, the patient was diagnosed with having lung-ETAC with a BRAF p.V600E mutation. Treatment with dabrafenib and trametinib initially resulted in positive results, with improvement in skin lesions and overall clinical condition. Nevertheless, approximately 6 months after, the disease had progression with new skin lesions reappearing.

**Conclusions:**

We reported a unique case of a patient with BRAF p.V600E-mutant lung-ETAC with metastatic skin lesions achieving complete cutaneous response after targeted treatment with dabrafenib and trametinib, highlighting the potential for targeted therapy in patients with lung-ETAC harboring a BRAF p.V600E mutation.

## Introduction

Enteric-type adenocarcinoma of the lung (lung-ETAC) is a rare lung cancer subtype, accounting only for 0.5% of cases and sharing similar morphologic and immunohistochemical features with colorectal cancer (CRC).^1^ Lung-ETAC diagnosis requires the expression of at least one enteric differentiation marker, including cytokeratin (CK) 20, CDX2, or MUC2, with the enteric pattern constituting at least 50% of the entire tumor. It is crucial to clinically exclude lung metastases from CRC to accurately identify lung-ETAC.^2^ Lung-ETAC typically displays early metastatic behavior and a higher likelihood of multiple metastases to several sites, such as the chest wall, abdomen, scalp, and brain.^3^ Rare cases of unusual metastatic sites (i.e., pancreas and skin) have also been reported, typically associated with poor prognosis.^3^^,^^4^ The treatment of advanced lung-ETAC includes chemotherapy, immunotherapy, radiotherapy, and targeted therapy, similarly to other NSCLC.

BRAF serine-threonine kinase p.V600E mutations are present in approximately 1% to 2% of lung adenocarcinomas. Nowadays, the combination of BRAF and MEK inhibitors represents the main first-line approach in advanced BRAF p.V600-mutant NSCLC.^5^ Interestingly, a previous report revealed that, among 13 patients with lung-ETAC, two carried a BRAF missense mutation.^6^

To the best of our knowledge, this case report is the first describing an enteric-type lung adenocarcinoma with diffuse skin lesions and a BRAF p.V600E mutation, undergone to a histologically confirmed complete cutaneous response to dabrafenib and trametinib.

## Case Presentation

A never-smoker 81-year-old man, with history of nonischemic heart disease, benign prostatic hyperplasia, and dyslipidemia, presented with dyschromic chest and abdomen skin lesions. In October 2022, a computed tomography (CT) scan revealed thickening in the retrohilar and paramediastinal regions of the lower right lobe, bilateral pleural effusion, carcinomatous lymphangitis, axillary, mediastinal, and retroperitoneal lymphadenopathies, and a solid pancreatic lesion with infiltration of the spleen and left adrenal gland.

**Figure 1:**
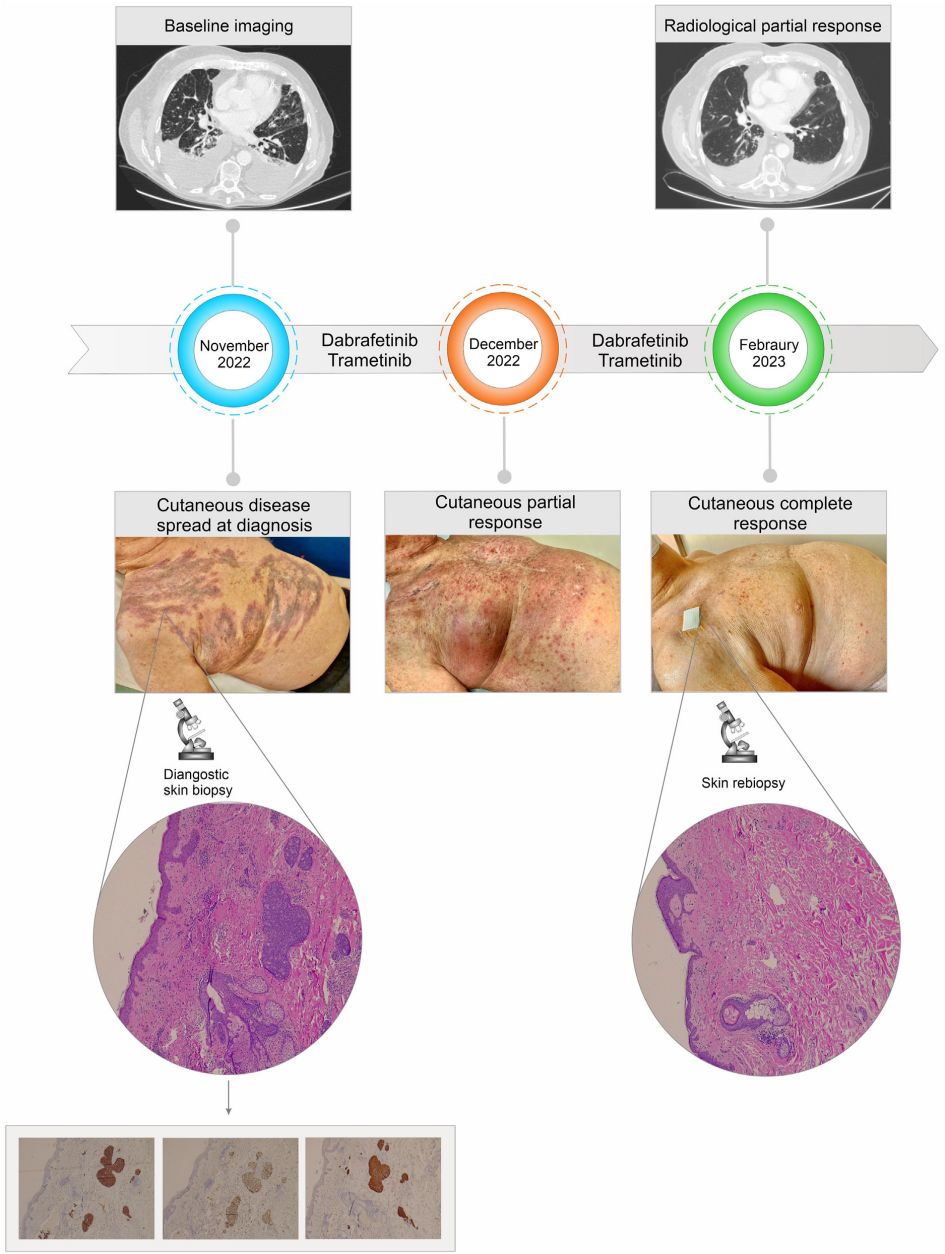
Upper panel: Timeline of the treatment course, with radiological (CT images) and clinical evidence (photos) of response. Lower panel: Histopathologic examination, at low magnification, reveals vascular neoplastic emboli in the dermis of chest skin. The tumor has a solid and cribriform architecture and enteric differentiation being positive for cytokeratin 7, CDX2, and villin. After 3 months of treatment, a new skin biopsy has been performed revealing a complete response and dermal fibrosis. CT, computed tomography.

At baseline, Eastern Cooperative Oncology Group performance status was 2, requiring continuous oxygen therapy. Physical examination results revealed dyschromic macules on the chest and right shoulder. A biopsy of the skin lesions was performed with the evidence of an enteric-pattern adenocarcinoma. Immunohistochemistry results revealed a strong positive staining for CK20, CDX2, CK7, and villin, with TTF1-negative staining (Fig. 1—lower panel). On the basis of immunohistochemical profile, a gastroscopy and a colonoscopy were performed, both of which reported negative results. A comprehensive molecular profiling using next-generation sequencing was performed, revealing the presence of BRAF p.V600E (rs113488022; variant allele frequency: 15%) and CTNNB1 S45F (rs121913409; variant allele frequency: 15%) mutations.

In November 2022, the patient initiated targeted therapy with dabrafenib (200 mg/d) and trametinib (1.5 mg/d) (Fig. 1—upper panel). At the first follow-up, after 3 weeks of treatment, there was an objective reduction of the skin lesions on the chest and abdomen. The patient experienced only G2 rash as a side effect. The clinical conditions gradually improved, without oxygen therapy. After approximately 3 months of treatment, a reevaluation CT scan revealed reduction in size of all known disease sites, with the complete resolution of the skin lesions, confirmed by a subsequent biopsy that resulted negative for cancer cells (Fig. 1—upper panel).

Nevertheless, at the restaging, approximately 6 months after the initiation of treatment, the CT scan revealed a multisite disease progression, along with the onset of novel skin lesions in the chest, but Eastern Cooperative Oncology Group performance status remained stable. Consequently, considering overall clinical condition, age, and patient and family wishes, he was addressed to best supportive care. The patient died in August 2023.

## Discussion

Lung-ETAC is a rare occurrence in lung cancer, and the coexistence of this histologic subtype with a BRAF p.V600E mutation adds an additional layer of rarity. Notably, a study has revealed that RAS alterations (KRAS and NRAS) and BRAF mutations were the most frequently observed aberrations, with frequencies of 31% and 11%, respectively.^7^

Regarding treatment strategies, no universal consensus was reached. Different options were evaluated, including platinum-based chemotherapy in combination with taxanes or immunotherapy^8^ or chemotherapy regimens frequently used for CRC, such as 5-fluorouracil, oxaliplatin, or irinotecan.^9^ No data are currently available on clinical management of *oncogene-addicted* lung-ETAC.

In our case, despite the patient’s advanced age and fragility, the use of BRAF and MEK inhibitors led to a good and noteworthy response, with a complete resolution of the cutaneous lesions, alongside the improvement in clinical conditions. This unique and intriguing cutaneous response to the treatment mirrors the behavior observed in other types of neoplasms, resembling the therapeutic effect noticed in other solid cancers, in primis in melanoma. Furthermore, the systemic evolution of the disease was consistent with that observed at cutaneous level.

To the best of our knowledge, no data are available on the activity and safety of target therapy in lung-ETAC, although preliminary evidence suggested a high frequency of *druggable* alterations.^7^ In light of this, our case report provides the first proof-of-principle of the therapeutical potential of *oncogene addiction* in lung-ETAC, confirming the feasibility of a target therapy even in an elderly and frail patient.

## Conclusion

Herein, we report a unique case of an elderly patient affected by an advanced lung-ETAC with cutaneous metastases harboring a BRAF p.V600E mutation, obtaining an impressive response and clinical benefit from a BRAF-MEK inhibitor combination. This case contributes to support further investigations about the druggability in clinical practice of this still-orphan-of-treatment subtype.

## CRediT Authorship Contribution Statement

**Marco Sposito:** Investigation, Data curation, Writing—original draft.

**Ilaria Mariangela Scaglione:** Investigation, Data curation, Writing.

**Serena Eccher:** Investigation, Data curation, Writing.

**Luca Pasqualin:** Investigation, Data curation, Writing.

**Alice Avancini:** Resources, Visualization.

**Chiara Colato:** Resources, Visualization.

**Paolo Rosina:** Resources, Visualization.

**Michele Simbolo:** Resources, Visualization.

**Anna Caliò:** Resources, Visualization.

**Aldo Scarpa:** Supervision.

**Michele Milella:** Supervision.

**Sara Pilotto:** Conceptualization, Writing—review and editing.

**Lorenzo Belluomini:** Conceptualization, Writing—review and editing.
